# Microdeletions and microduplications linked to severe congenital disorders in infertile men

**DOI:** 10.1038/s41598-023-27750-w

**Published:** 2023-01-11

**Authors:** Triin Kikas, Anna Maria Punab, Laura Kasak, Olev Poolamets, Vladimir Vihljajev, Kristjan Pomm, Mario Reiman, Stanislav Tjagur, Paul Korrovits, Margus Punab, Maris Laan

**Affiliations:** 1 Institute of Biomedicine and Translational Medicine, University of Tartu, Ravila 19, 50411 Tartu, Estonia; 2 Andrology Clinic, Tartu University Hospital, L. Puusepa 1a, 50406 Tartu, Estonia; 3 Institute of Clinical Medicine, University of Tartu, L. Puusepa 8, 50406 Tartu, Estonia

**Keywords:** Medical genetics, Disease genetics, Male factor infertility

## Abstract

Data on the clinical validity of DNA copy number variants (CNVs) in spermatogenic failure (SPGF) is limited. This study analyzed the genome-wide CNV profile in 215 men with idiopathic SPGF and 62 normozoospermic fertile men, recruited at the Andrology Clinic, Tartu University Hospital, Estonia. A two-fold higher representation of > 1 Mb CNVs was observed in men with SPGF (13%, n = 28) compared to controls (6.5%, n = 4). Seven patients with SPGF were identified as carriers of microdeletions (1q21.1; 2.4 Mb) or microduplications (3p26.3, 1.1 Mb; 7p22.3-p22.2, 1.56 Mb; 10q11.22, 1.42 Mb, three cases; Xp22.33; 2.3 Mb) linked to severe congenital conditions. Large autosomal CNV carriers had oligozoospermia, reduced or low-normal bitesticular volume (22–28 ml). The 7p22.3-p22.2 microduplication carrier presented mild intellectual disability, neuropsychiatric problems, and short stature. The Xp22.33 duplication at the PAR1/non-PAR boundary, previously linked to uterine agenesis, was detected in a patient with non-obstructive azoospermia. A novel recurrent intragenic deletion in testis-specific *LRRC69* was significantly overrepresented in patients with SPGF compared to the general population (3.3% *vs*. 0.85%; χ^2^ test, OR = 3.9 [95% CI 1.8–8.4], *P* = 0.0001). Assessment of clinically valid CNVs in patients with SPGF will improve their management and counselling for general and reproductive health, including risk of miscarriage and congenital disorders in future offspring.

## Introduction

Male factor infertility is a prevalent health condition (5–10% of men) with broad etiologies, clinical and social consequences^1^. Known genetic causes (e.g., 47, XXY karyotype) explain < 10% of male infertility cases, whereas every second patient remains idiopathic^2^. Copy number variants (CNVs) refer to locus deletions or duplications that span from single genes to large genomic segments. CNVs may modulate the process of spermatogenesis through altered gene dosage effect, impaired homologous recombination, and/or genomic instability leading to errors in chromosomal segregation. So far, data on the role and clinical validity of CNVs predisposing to male infertility is limited^3^. The only CNVs included in the diagnostic workup of men with spermatogenic failure (SPGF) are sporadic microdeletions encompassing three *Azoospermia Factor regions* (*AZFa, AZFb* or *AZFc*) located in the Y chromosome and mostly leading to complete lack of mature sperm^2^. Recently, a novel Y-haplogroup specific inversion was described that predisposes to recurrent *AZFc* deletions and consequently, to SPGF^4^. Also some rare autosomal and X-linked CNVs have been confidently shown to cause male infertility phenotypes, such as heterozygous loss of *WT1* and congenital genitourinary disorders^5^, hemizygous deletions of *TEX11* and meiotic arrest^6^, biallelic loss of *DPY19L2* and globozoospermia^7^. A multi-center study of patients with azoospermia has reported rare non-recurrent deletions in the *DMRT1* genomic region involved in sex development^8^.

A decade ago, two seminal studies profiling genome-wide CNVs in patients with SPGF reached concordant results that the average number and total load of DNA gain/loss per genome was similar to fertile men and the general population^8,9^. The few reports published on this topic across 10 years have not identified any additional replicated candidate infertility-linked CNVs^8–13^. Therefore, more investigations are needed to fill the gaps in this underexplored field.

This study aimed to characterize the genome-wide profile of CNVs in 215 idiopathic infertility subjects with variably reduced spermatogenic output, and 62 normozoospermic fertile men as controls. As the main objective, the carriership of large deletions and duplications was analyzed under the hypothesis that increased chromosomal instability and/or altered dosage of multiple genes may predispose to SPGF. As a secondary objective, rare recurrent CNVs and gains/losses of genes implicated in monogenic forms of male infertility were explored.

## Materials and methods

### Recruitment and formation of the study cohort

The study was approved by the Ethics Review Committee of Human Research of the University of Tartu, Estonia (permission no. 74/54, last amendment 288/M-13) and complied with the Helsinki Declaration. Written informed consent was obtained from each study subject prior to recruitment. Study participants were recruited to the Estonian Andrology (ESTAND) cohort^2^ and the material was collected at the Andrology Clinic, Tartu University Hospital (AC-TUH), Estonia. AC-TUH represents the primary and referral center managing > 90% of all male infertility cases in the country. SPGF (total sperm count < 39 × 10^6^ per ejaculate) was defined according to the World Health Organization^14^. Stratification to severe (SO, sperm count > 0 and < 10 × 10^6^) and moderate oligozoospermia (MO, 10–39 × 10^6^) was implemented as described^2^. Non-obstructive azoospermia (NOA) refers to lack of sperm in the ejaculate due to primary SPGF.

The genome-wide CNV profile was generated and analyzed for 277 ESTAND participants (median age 33, range 18–58 years; Table 1). The study group was formed of 215 patients with idiopathic SPGF (NOA n = 73; SO, n = 84; MO, n = 58) and 62 normozoospermic fertile men (NORM)^2,15^. The known causes of male infertility were excluded (Supplementary Methods). All men had passed identical clinical assessment and phenotyping using the established andrological pipeline and standard protocols at the AC-TUH. Since none of the men in the NORM group as well as 48 men with SPGF (n = 11 NOA, 14 SO, 23 MO) had undergone the standard cytogenetic analysis, all samples were evaluated for sex chromosome abnormalities using the genome-wide SNP genotyping data and the established approach^16^.

**Table 1:** Clinical parameters of 277 study subjects.

Parameter^a^	Non-obstructive azoospermia (NOA)	Severe oligozoospermia (SO)	Moderate oligozoospermia (MO)	Normozoospermic fertile men (NORM)^b^
Subgroup size (n)	73	84	58	62
Age (years)	33.7 (21.6–58.0)	35.6 (18.1–55.4)	32.4 (20.0–57.8)	31.0 (22.0–53.0)
Height (cm)	180 (169–197)	178 (166–198)	181.5 (162–210)	180 (171–193)
Weight (kg)	85.9 (54.3–127.7)	85.2 (55.7–125.8)	86.2 (59.7–123.0)	80.3 (62.0–107.4)
BMI (kg/m2)	26.1 (18.7–35.7)	26.9 (18.4–37.8)	25.7 (18.0–34.4)	25.0 (19.7–34.2)
Total testis volume (ml)	27 (7–49)	28 (7–55)	28 (18–56)	47.5 (34–100)
Semen volume (ml)	3.4 (0.5–8.9)	3.6 (0.6–8.2)	4.0 (1.4–9)	4.1 (1.8–9.4)
Total sperm count (× 10^6^/ ejaculate)	0 (0–0)	2.4 (0.004–9.9)	21.3 (10–37.8)	430.3 (49.5–1540)
Sperm concentration (× 10^6^/ml)	0 (0–0)	0.9 (0.001–14)	5.0 (1.8–19)	115 (15–355)
Progressive A + B motility (%)^c^	n/a	14.5 (0–70)	30 (0–60)	55 (41–84)
Sperm with normal morphology (%)^d^	n/a	0 (0–12)	2 (0–12)	14 (2–27)^e^
FSH (IU/l)	24.9 (4.8–70.5)	17.4 (2.7–41.1)	13.4 (4.1–30.5)	3.2 (0.8–6.8)
LH (IU/l)	8.3 (2.7–27.8)	5.9 (2.2–16.4)	4.8 (2.5–14.6)	3.4 (1.5–6.8)
Total testosterone (nmol/l)	14.0 (1.0–45.0)	16.5 (3.5–46.0)	16.5 (5.8–41.1)	16.7 (9.1–30.1)

^a^Median (range) is shown for each parameter.

^b^Normozoospermic (total sperm count > 39 × 10^6^/ ejaculate) partners of pregnant women.

^c^WHO normal range 30–75%^14^.

^d^WHO normal range 4–39%^14^.

^e^61/62 subjects in the NORM subgroup had normal sperm morphology (10–27% of sperm).

n/a, not applicable.

All participants were of white European ancestry and living in Estonia.

### Calling and analysis of CNVs

Blood genomic DNA was genotyped using Illumina HumanOmniExpress-24-v1.0/v1.1 BeadChips at the institutional genotyping core facility (https://genomics.ut.ee/en/genomics-core-facility). CNVs were called based on 705,754 SNPs present at the same genomic locations on both genotyping array versions. The established pipeline for autosomal CNV calling was used^17,18^, based on parallel implementation of three CNV prediction algorithms – QuantiSNP v2.3^19^, GADA (Genome Alteration Detection Analysis), and CNstream^20^ (Supplementary Methods). The HD-CNV algorithm^21^ was implemented to identify overlapping CNVs called by alternative prediction tools. A criterion of 40% reciprocal overlap in the predicted region (minimal length of 100 bp) for the same type of event (deletion or duplication) was used to define confident CNVs. All CNVs called by at least two algorithms for the same individual were included in the final list (Supplementary Table S1). A large microduplication at 7p22.3-p22.2 (Case SO1) was validated by array-CGH and the carriership of an *LRRC69* intragenic deletion among 215 SPGF cases was confirmed by TaqMan qPCR (Supplementary Methods).

X chromosome CNVs were detected using the Illumina Genomestudio 2.0.5 built-in CNV analysis tool with default settings (cnvPartition CNV Analysis Plugin v3.2.0), as algorithms used for autosomal CNV calling tend to perform with low confidence for sex chromosome analysis^8^ (Supplementary Methods). To avoid spurious predictions, only CNVs calls > 10 kb were considered (Supplementary Table S1).

Human genome databases (DECIPHER^22^, The Database of Genomic Variants^23^, OMIM, https://omim.org) were utilized to extract phenotype and breakpoint data for previously mapped CNVs in the regions of interest and their reported prevalence in the general population. Clinical significance of the highlighted large (> 1 Mb) autosomal and X chromosome CNVs was assessed according to the American College of Medical Genetics and Genomics guidelines^24^. Recent literature reviews were used to assemble the list of genes implicated in monogenic forms of male infertility^25,26^.

### Statistical analysis

Statistical analyses were performed using the R Statistical Software version [3.5.4] (http://www.r-project.org/). Two-tailed Student t-test was used to assess the differences between clinical subgroups in the size and count of CNVs, and the χ^2^ test to compare the distribution of deletions and duplications. The nominal *P* value < 0.05 was considered statistically significant.

Enrichment of the *LRRC69* deletion in SPGF was tested compared to the CNV dataset of 45,390 population-based subjects^27,28^ recruited to the Estonian Biobank (Supplementary Methods).

## Results

### Profile of autosomal deletions and duplications identified in the study group

**Figure 1:**
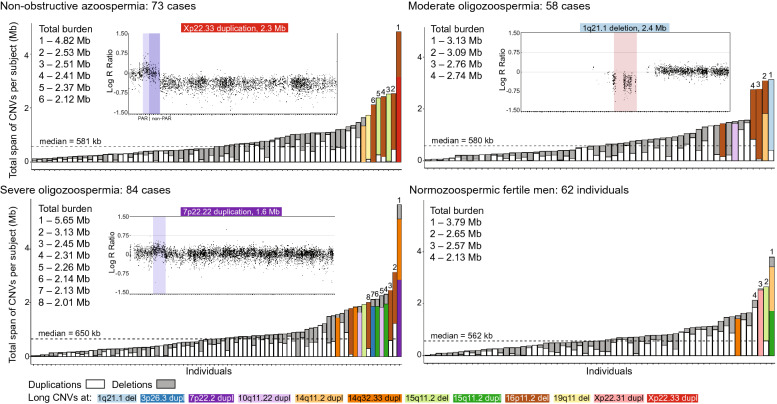
The burden of copy number variants in the subgroups based on spermatogenic output. Y-axis shows the cumulative burden of all identified autosomal and X-chromosomal CNVs for every study subject (X-axis). Deletions and duplications larger than 1 Mb are indicated with color coding, and example variants are visualized with Genomestudio 2.0.5 (Illumina Inc.). Subjects with total burden of CNVs spanning over 2 Mb are indicated by numbering. Dotted lines refer to the median total span of CNVs in each subgroup.

Across 277 study subjects (Table 1), 2026 autosomal CNVs were identified in total (median size 33 kb, range 185 bp–2.4 Mb; Table 2, Supplementary Table S1). Losses were mapped to 697 and gains to 512 unique loci (Fig. S1-2). The median length of a duplication exceeded over fourfold the median span of a deletion (86.2 *vs.* 19.6 kb Student t-test, *P* = 2.4 × 10^–17^). The most extensive rearrangements were detected on chromosome 16 (mean 911 bp/ Mb per subject, Fig. S3). No significant differences were observed in the median number and load of CNVs per subject among subgroups stratified by spermatogenic output (Fig. 1, Table 2, Fig. S4). Trends for a longer cumulative span of deletions (median 191 *vs.* 160 kb; *P* < 0.05) and a shorter span of duplications (292 *vs.* 399 kb; *P* < 0.05) were observed in patients with NOA compared to normozoospermic men. The summary data of genome-wide CNVs was consistent with previous reports^8–11^.

**Table 2:** Summary statistics of identified autosomal deletions and duplications^a^.

Parameter	All (n = 277)	NOA (n = 73)	SO (n = 84)	MO (n = 58)	NORM (n = 62)
All CNVs (n)	2,026	526	610	435	455
All deletions (n)	1,279	343	375	280	281
All duplications (n)	747	183	235	155	174
Size of CNVs (kb)	33.1 (0.2–2413)	31.7 (0.3–2397)	34.7 (0.2–1856)	32.4 (0.3–2413)	37.6 (0.3–1856)
Size of deletions (kb)	19.6 (0.2–2413)	14.8 (0.3–2397)	20.7 (0.2–1856)	18.1 (0.3–2413)	22.5 (0.3–1856)
Size of duplications (kb)	86.2 (0.4–1856)	82.1 (3.8–1089)	85.4 (0.4–1856)	81.7 (0.5–1420)	112.7 (1.7–1856)
CNVs per individual (n)	7.3	7.2	7.3	7.4	7.3
Cumulative span of all CNVs per subject (kb)	579 (24–5033)	496 (115–2524)	644 (31–5033)	576 (55–3135)	562 (24–3795)
Deletions per subject (n)	4.6	4.7	4.5	4.8	4.5
Cumulative span of deletions (kb)	173 (0.9–2724)	191 (4.8–2482)*	141 (0.9–1917)	186 (1.1–2724)	160 (8.3–2084)
Duplications per subject (n)	2.7	2.5	2.8	2.6	2.8
Cumulative span of duplications per subject (kb)	329 (2.2–4492)	292 (29–1250)*	418 (5.0–4492)	223 (2.2–1819)	399 (20–3411)
> 1 Mb CNVs in total (n)	33	8	14	7	4
> 1 Mb deletions (n)	18	7*	5	5	1
> 1 Mb duplications (n)	15	1	9	2	3

Data shown as median (range) unless stated otherwise. CNV burden for each analyzed subject is shown on Fig. 1

^a^X chromosome CNVs were analyzed using an alternative approach and respective summary statistics are presented in Supplementary Table S3.

*NOA *vs* NORM, nominal Student t-test *P* < 0.05.

*NORM* Normozoospermic fertile men, *MO* Moderate oligozoospermia, *NOA* Non-obstructive azoospermia, *SO* Severe oligozoospermia.

Over 1 Mb long autosomal deletions (n = 18; mapping to four regions) and duplications (n = 17; eight regions) were detected in 32 of 277 (11.6%) study subjects (Fig. 1, Tables 3 and 4, Supplementary Table S2). A trend for overrepresentation of large deletions in men with SPGF (17 of 215) compared to NORM subjects (one of 62) was observed (7.9% vs 1.6%; χ^2^ test, *P* = 0.08). The highest proportion of large duplications was identified in patients with SO (9 of 84; 10.7%).

**Table 3:** Large (> 1 Mb) autosomal and X chromosome deletions and duplications identified among analyzed individuals.

CNV ID^a^	Size	Carriers (n)				CNV region features^c^	Published literature	
Effect^b^	hg19	NOA	SO	MO	NORM		Clinical phenotypes and inheritance	Patients in this study
1q21.1 del Pathogenic	2.4 Mb	0	0	1	0	28 genes, including dominant *PIAS3*, *ANKRD34A, PRKAB2*, *BCL9, RBM8A; GJA8* has enriched protein expression in spermatocytes and early spermatids	1q21 microdeletion syndrome with recurrent breakpoints (OMIM#612,474, ORPHA:250,989): mild facial dysmorphisms, microcephaly, DD, ID and congenital cardiac, ocular, skeletal, and psychiatric or behavioral conditions; variable penetrance including asymptomatic carriers; de novo or inherited from either of the parents^29,30^	no congenital or chronic health conditions documented in health records, except for SPGF
15q11.2 del Likely benign	2.4 Mb	2	1	0	1	pericentromeric; *OR4* gene cluster; primate-specific and testis-enriched *GOLGA6L6* (early spermatids) and *LINC02203* genes, and the *POTEB* gene cluster	no established links to clinical phenotypes	no congenital or chronic health conditions except for SPGF in 3 of 4 men
16p11.2 del VUS	2.0 Mb	4	4	4^d^	0	pericentromeric; epididymis and testis-specific *TP53TG3* gene cluster with enriched expression in spermatocytes, early and late spermatids	no established links to clinical phenotypes	no congenital or chronic health conditions except for SPGF
19q11 del VUS	1.4 Mb	1	0	0	0	pericentromeric; no protein coding genes	no established links to clinical phenotypes	no congenital or chronic health conditions except for NOA
All del		7	5	5	1			
7p22.2 dupl VUS	1.6 Mb	0	1^e^	0	0	*AMZ1, GNA12, CARD11, SDK1*	rare non-recurrent 7p22.3-p22.2 microduplication syndrome with variable breakpoints: facial dysmorphisms, short stature, DD, ID, asthma, myopia; other conditions; de novo or due to unbalanced chromosome translocations ^31^	mild ID, problems with cognitive performance, residual schizophrenia; diagnosed with joint disorders; short stature (170 cm)
10q11.22 dupl VUS	1.4 Mb	0	2	1	0	*ANTXRL* is a testis-specific gene expressed in early and late spermatids; *PTPN20* has enhanced expression in spermatocytes and spermatids Other: *ANXA8L1, GDF10, GDF2, GPRIN2, NPY4R, RBP3, SYT15, ZNF488*	rare non-recurrent duplications with variable breakpoints; DD, ID, microcephaly, childhood-onset epilepsy, seizures, autistic features and other health conditions; de novo or parental karyotype data unavailable^32,33^	asymptomatic to typical 10q11.22 dupl phenotype; one patient has been treated for various dermatological conditions
3p26.3 dupl VUS	1.1 Mb	0	1	0	0	*CNTN4*	rare non-recurrent microduplication syndrome with variable breakpoints: DD, ID and autism spectrum disorder; incomplete penetrance*; *de novo or inherited from healthy parents ^34^	asymptomatic to typical 3p26.3 dupl phenotype; no congenital or chronic health conditions except for SPGF; short stature (167 cm)
14q32.33 dupl VUS	1.2 Mb	0	3^e^	0	1	subtelomeric; *IGH* gene cluster prone to rearrangements	rare 14q32.33 terminal duplications with variable breakpoints and expressivity, e.g. low birth weight, hypotonia, facial dysmorphisms, DD, ID and other health conditions; de novo or due to unbalanced chromosome translocations ^35^	asymptomatic to typical 14q32.33 dupl phenotype; in two cases, no congenital or chronic health conditions except for SPGF
14q11.2 dupl Likely benign	1.1 Mb	1	0	1^d^	1f.	pericentromeric; *POTEG, POTEM, OR11* and *OR4* gene families	no established links to clinical phenotypes	no congenital or chronic health conditions except SPGF in 2 of 3 men
15q11.2 dupl Likely benign	1.9 Mb	0	2	0	1f.	pericentromeric; see 15q11.2 del	no established links to clinical phenotypes	no congenital or chronic health conditions except SPGF in 2 of 3 men
Xp22.33 dupl VUS	2.3 Mb	1	0	0	0	encompasses the boundary of PAR1 (*DHRSX, ZBED1, CD99, XG*) and non-PAR regions (*GYG2, ARSD, ARSL, ARSH, ARSF, MXRA5, PRKX*)	previously described in one female with Mayer–Rokitansky–Küster–Hauser syndrome II; she had vaginal and uterine agenesis, horseshoe kidney, hypoacusis, tachycardia, osteoporosis; parental karyotype data unavailable^36^ The effect in men is currently unknown	no congenital or chronic health conditions except for SPGF
Xp22.31 dupl Benign	1.7 Mb	0	0	0	1	*PUDP, STS, PNPLA4; VCX* is a testis-enriched gene that is expressed in spermatocytes and spermatogonia	rare non-recurrent duplications with variable breakpoints; no association to clinical phenotypes in the UK biobank study; de novo or inherited from either of the parents ^37^	no congenital or chronic health conditions
All dupl		2	9	2	4			

^a^Exact genomic coordinates of identified large CNVs are detailed in Table 4 and Supplementary Table S2.

^b^Assessment based on the recommendations of the American College of Medical Genetics and Genomics^24^.

^c^Testicular expression of the encompassed genes was assessed based on the Human Protein Atlas; https://www.proteinatlas.org.

^d^One moderate oligozoospermia case carried 16p11.2 microdeletion and 14q11.2 microduplication.

^e^One severe oligozoospermia case carried 7p22.2 and 14q32.33 microduplications.

^f^One normozoospermic fertile man carried 14q11.2 and 15q11.2 microduplications.

*DD* Developmental delay, *del* Deletion, *dupl* Duplication, *ID* Intellectual disability, *MO* Moderate oligozoospermia, *NOA* Non-obstructive azoospermia, *NORM* Normozoospermic fertile men, *PAR* Pseudoautosomal region, *SO* Severe oligozoospermia, *VUS* Variant on uncertain significance.

**Table 4:** Detailed clinical features of undiagnosed carriers of clinically relevant large microdeletion or microduplications.

Case ID	CNV ID	CNV data (hg19)			General parameters				Testicular and sperm parameters				Hormonal parameters		
		Start (bp)	End (bp)	Size (Mb)	Age (yrs)	H (cm)	W (kg)	BMI (kg/m2)	TTV (ml)	Semen vol (ml)	Sp conc	Sp count	FSH (IU/l)	LH (IU/l)	Total T (nmol/l)
MO1	1q21.1 del	145,413,715	147,826,789	2.41	32	178	78	24.6	28	7.2	5.1	36.7	13.2	4.1	21.8
SO1^a^	7p22.2 dupl	2,727,337	4,283,564	1.56	35	170	88	30.4	23	2.9	0.1	0.2	13.7	5.5	9.8
SO2	3p26.3 dupl	1,571,508	2,669,708	1.10	41	167	84	30.3	22	1.7	1.5	2.6	14.7	4.6	33.8
SO3	10q11.22 dupl	46,283,686	47,703,869	1.42	30	182	95	28.6	27	2.4	1.0	2.4	22.9	13.8	22.2
MO2	10q11.22 dupl	46,283,686	47,703,869	1.42	33	178	80	25.2	25	3.0	7.2	21.3	24.2	4.3	10.6
SO4	10q11.22 dupl	47,101,942	48,318,619	1.22	43	178	102	32.3	23	3.7	2.0	7.4	22.8	5.1	3.5
NOA9	Xp22.33 dupl	1,853,035	4,151,086	2.30	28	191	90	24.7	45	5.8	0	0	21.1	3.5	17.4

^a^The case also carried a large CNV at 14q32.33 which is likely to be a benign change (detailed in Supplementary Table S2).

*FSH* Follicle stimulating hormone, *H* Height, *LH* Luteinizing hormone, *n.d.* not documented, *Sp* *conc* Sperm concentration (× 10^6^/ml), *Sp count* Sperm count (× 10^6^/ per ejaculate), *T* Testosterone, *TTV* Total testis volume, bitesticular (left + right), *W* Weight.

### Patients with SPGF carried microdeletions and microduplications linked to severe congenital syndromes

Six of 215 cases with SPGF (2.8%) carried large autosomal microdeletions (1q21.1) or microduplications (3p26.3, 7p22.3-p22.2, 10q11.22) linked to severe congenital syndromes in patients with developmental delay (DD), intellectual disability (ID), facial dysmorphism, and/or other health conditions (Fig. 1; Table 3). The recurrent 1q21.1 microdeletion is classified as pathogenic, whereas the non-recurrent microduplications currently represent variants of uncertain significance due to low number of characterized subjects and variable breakpoints.

For the carrier of a 1.56 Mb 7p22.3-p22.2 microduplication^31^ (case SO1: age 35 years, sperm count 0.2 × 10^6^/ejaculate, bitesticular volume 23 ml, FSH 13.7 IU/L; Table 4), retrospective clinical records revealed mild ID, behavioral and cognitive problems, residual schizophrenia, and diagnosis of a joint disorder. He also presented obesity (BMI > 30) and height (170 cm) that was over 10 cm below the average male height in the Estonian population (181–182 cm)^2^. The patient SO1 also carried a subtelomeric non-recurrent 14q32.33 microduplication (1.2 Mb), recently linked to DD/ID^35^. The carriership of both large CNVs was externally validated by aCGH (Methods, Fig. S5a). Neither 7p22.3-p22.2 nor 14q32.33 microduplication encompassed genes that were apparent candidates for SPGF. Other subjects with the 14q32.33 microduplication (SO10, SO11, and NORM2) exhibited no congenital abnormalities during their andrological workup (age 30–42 years; Supplementary Table S2).

The carriers of the recurrent 1q21.1 microdeletion^29,30^ (case MO1; 2.6 Mb; OMIM#612474; Fig. 1), and non-recurrent microduplications at 3p26.3^34^ (case SO2; 1.1 Mb) and 10q11.22^32,33^ (cases SO3, SO4, MO2; 1.2–1.4 Mb) presented moderate or severe oligozoospermia (Fig. S5b). During infertility workup (age 30–43 years), no typical phenotypic conditions linked to these variants were reported. However, all these men had reduced or low-normal bitesticular volume (22–28 ml vs. median 47.5 ml in NORM subjects, Table 1) and high FSH (13.2–24.2 IU/l), suggesting congenital testicular failure. All patients with autosomal microduplications were overweight (BMI 25–30; two cases) or obese (BMI > 30; three cases), and also the 1q21.1 microdeletion carrier had a high BMI (24.6). Additionally, the patient with the 3p26.3 microduplication presented a short stature (167 cm) compared to typical Estonian men.

### A NOA patient with a 2.3 Mb X chromosome duplication encompassing the PAR1/non-PAR boundary

X chromosome CNVs (median 128.8 kb; range 14–2298 kb) were mapped to 31 unique regions (Supplementary Tables S1, S3). No enrichment was identified in men with SPGF compared to NORM subjects.

Patient NOA9 carried a 2.3 Mb duplication at Xp22.33 (Fig. 1, Tables 3, 4, Supplementary Table S2). Interestingly, he presented tall stature (191 cm) and a high FSH level (21.1 IU/L, aged 28 years) that are also typical to patients with Klinefelter syndrome (mostly presenting NOA) caused by extra copies of the X chromosome^38^. However, his bitesticular volume (45 ml) and testosterone level (17.4 nmol/l) were normal. The identified DNA gain encompasses the boundary between the pseudoautosomal (PAR1, ~ 1 Mb) and non-PAR regions (~ 1.3 Mb) and represents a rearrangement-prone locus affecting recombination between the X and Y chromosomes^39^. This variant has been reported in a female patient with vaginal and uterine agenesis^36^.

One recurrent X chromosome variant, a ~ 90 kb deletion encompassing *ZNF630* at Xp11.23, was detected only in cases with SPGF (one NOA, two SO). Although its allele frequency in this study and in a large multicenter dataset of cases with NOA^8^ was similar (1.4% and 1.6%, respectively), this deletion has been reported with a 1.2% prevalence in the general population (gnomAD SVs v2.1: variant: DEL_X_186083) and therefore, is an unlikely major contributor to SPGF.

The rest of the X chromosome CNVs were mapped to either intergenic loci (22 of 43 CNVs, 51%), spanned duplicated gene families, represented common variants in the general population, and/or did not include any strong candidate genes for SPGF. Also, a large gain at Xp22.31 (1.7 Mb) identified in the NORM4 subject was evaluated as benign in the UK Biobank dataset of 421,413 individuals^37^.

### A large deletion in the pericentromeric region of 16p11.2 as a candidate risk factor for SPGF

Large CNVs in pericentromeric regions (1.1–2.4 Mb) were detected in 21 of 277 study subjects (7.6%), including 17 deletions at 15q11.2, 16p11.2, or 19q11.2; and six duplications at 14q11.2 or 15q11.2 (Fig. 1, Table 3, Supplementary Table S2). None of the men carrying large CNVs in pericentromeric regions presented severe congenital or chronic health conditions apart from SPGF.

**Figure 2:**
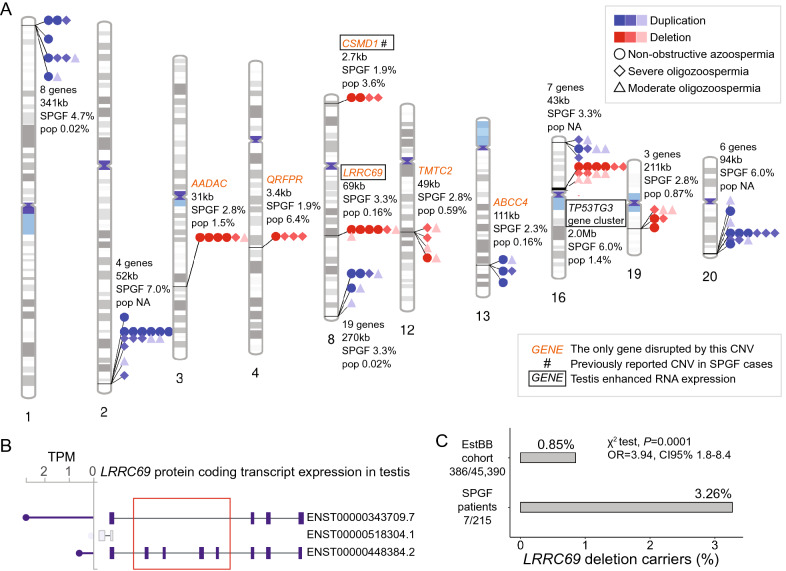
Recurrent CNVs in men with spermatogenic failure. (**A)** Chromosomal distribution of recurrently detected deletions and duplications among 215 cases with spermatogenic failure (SPGF). The highlighted 13 CNVs represent the same type of copy number change (seven deletions, six duplications) encompassing protein-coding gene(s) and detected in at least four cases with SPGF and no normozoospermic fertile men (NORM). The number of encompassed genes, and the length and frequency of each variant in subjects with SPGF compared to the general population (pop; sources: DGV Gold^23^, DECIPHER^22^) are shown. Recurrent CNVs exceeding 1 Mb or disrupting only a single gene are highlighted. Intronic *CSMD1* deletions (indicated by #) have been proposed as candidate contributors to male infertility^8^, but in this study the detected prevalence in cases with SPGF was lower than reported in the general population. Details are presented in Supplementary Table S4. (**B)** The major protein-coding transcripts of the *LRRC69* gene expressed in the human testis according to the GTEx database^40^. The identified *LRRC69* partial deletion is shown with a red box. Genomic coordinates of the minimal deleted region (52,375 bp) are chr8:92,128,840–92,181,214 (hg19). TPM, transcripts per million. (**C)** Statistically significant association between *LRRC69* intragenic deletions among cases with SPGF compared to population-based participants in the Estonian Biobank (EstBB) cohort^27,28^. A χ^2^ test was used to test the difference between the two groups.

A recurrent deletion in the pericentromeric region of 16p11.2 (2 Mb) was detected in 12 of 215 SPGF patients (median sperm count 2.9 × 10^6^ / ejaculate) but was not identified in NORM subjects (Fischer exact test, *P* = 0.07). The frequency observed in cases with SPGF exceeded the reported population prevalence fourfold (5.6% *vs.* 1.4%; Fig. 2a, Supplementary Table S4). The 16p11.2 deletion encompasses the hominid-specific *TP53TG3* cluster comprised of six duplicated genes with enriched expression in the epididymis, spermatocytes, early and late spermatids.

Deletions in pericentromeric regions of 19q11 (1.4 Mb, case NOA8) and 15q11.2 (1.9–2.4 Mb, NOA6-7, SO12, NORM3) had no apparent link to SPGF. The subject NORM1 carried duplications at 14q11.2 (1.1 Mb) and 15q11.2 (1.9 Mb), excluding these CNVs as likely risk factors for SPGF.

### A recurrent partial deletion of the testis-specific *LRRC69* gene is significantly associated with SPGF

Six smaller autosomal deletions (median 41.0 kb, range 1.9–191.6 kb) and six duplications (median 50.3 kb, range 16.5–341.3 kb) (all heterozygous) were identified that were carried by four or more patients with SPGF but not by any NORM subjects (Fig. 2a, Supplementary Table S4). No recurrent CNVs were overrepresented in a specific SPGF subgroup.

A heterozygous partial *LRRC69* gene deletion (52–69 kb) that removes four exons of its alternative transcript (ENST00000448384.2) was detected and validated using a locus-specific assay in seven patients with SPGF, including four cases with NOA (Methods, Fig. 2b, Supplementary Table S5). This previously undescribed testis-enhanced gene is expressed in early and late spermatids^41^ but there is no data on the phenotypic consequences of its reduced dosage. A highly significant enrichment of the *LRRC69* deletion among cases with SPGF compared to population-based participants in the Estonian biobank (EstBB)^27,28^ was observed (7/215 *vs.* 386/45,390; 3.3% *vs.* 0.85%; χ^2^ test, OR = 3.9 [95%CI 1.8–8.4], *P* = 0.0001; Fig. 2c, Supplementary Table S6).

Other gene deletions were ranked as low priority due to their high population prevalence (*AADAC, CSMD1, QRFPR* loci; 1.5–6.4%) or unlikely major involvement of lost gene copies in SPGF (*TMTC2, PSG* cluster). All but one (*ABCC4*) recurrent duplications were mapped to subtelomeric regions with no present evidence that increased dosage of encompassed genes would affect spermatogenesis.

### CNVs involving genes linked to monogenic male infertility are not enriched in cases with SPGF

Nine heterozygous deletions (three independent loci) and two duplications (two loci) were identified encompassing genes linked to monogenic infertility (Supplementary Table S7). Most involved genes were linked to disorders with autosomal recessive inheritance. One patient with NOA carried four copies of *PLXNA1*, recently linked to autosomal dominant hypogonadotropic hypogonadism^42^. As the patient’s hormonal profile (FSH: 14 IU/l) did not match this condition, the effect of increased gene dosage was unclear.

## Discussion

This study analyzed the genome-wide CNV profile in 215 patients with idiopathic SPGF and 62 normozoospermic fertile men. A considerable number of patients with SPGF (n = 28; 13%) carried large deletions and duplications spanning over 1 Mb, whereas the respective carrier frequency in the normozoospermic fertile men was two times lower (n = 4, 6.5%). The proportion of large CNVs in infertile men is in a similar range as reported for patients with autism^43^ or DD^44^. In comparison, a study of 7,877 Estonian Biobank participants identified only 2% of subjects as carriers of > 1 Mb CNVs^27^.

As the main outcome of this study, seven patients with SPGF (~ 3.3%) were identified as undiagnosed carriers of microdeletions (1q21.1) or microduplications (3p26.3, 7p22.3-p22.2, 10q11.22, Xp22.33) linked to severe congenital developmental conditions (Table 3). This is comparable to the prevalence of Y chromosome *AZFa-c* microdeletions (2–10%) currently included in the diagnostic pipeline for SPGF^2,45,46^. Clinical data of patients with large autosomal DNA gains/losses are supportive to congenital testicular maldevelopment (bitesticular volume < 30 ml, FSH > 12 IU/l) as their primary cause of SPGF (Table 4, Supplementary Table S2). Case SO2 (total sperm count 0.2 × 10^6^) with the non-recurrent 7p22.3-p22.2 microduplication also presented mild ID, neuropsychiatric problems, and short stature. To our knowledge, this is the first clinical description of an adult subject with this variant, so far reported in pediatric patients with a severe syndromic DD phenotype^31^. One patient with SPGF was identified as a carrier of the recurrent 1q21.1 microdeletion spanning 2.4 Mb (Fig. 1). The prevalence of undiagnosed cases carrying this large CNV has been reported 1/2,626 in the Estonian population-based study^27^. The 1q21.1 microdeletion is listed in the DECIPHER^22^ database of developmental disorders and characterized by incomplete penetrance and variable expressivity, including infertility and cryptorchidism in some male carriers^29,30^. Unfortunately, general health data of the case in our study was unavailable. Inheritance from healthy parents and incomplete penetrance have also been evidenced for the 3p26.3 microduplication^34^, whereas other identified non-recurrent microduplications have been reported mostly as de novo events with no penetrance estimates (Table 3). Non-recurrent microduplications at 10q11.22 have been described in singleton cases with pediatric-onset epilepsy and ID^32,33^, but this study identified three carriers among SPGF cases. Their andrological data resembled the phenotype of the 1q21.1 deletion carrier – oligozoospermia, lower bitesticular volume compared to controls, increased FSH and BMI (Table 4). The variant spans two candidate genes with testis-specific (*ANTXRLI,* restricted to spermatids) or -enhanced (*PTPN20,* spermatocytes and spermatids) expression. The case with NOA carrying the Xp22.33 duplication spanning the PAR1/non-PAR boundary had normal testes size, suggesting a primary defect in the process of spermatogenesis per se. As the amplified region involved no apparent candidate genes, a structural effect disturbing recombination between X and Y is a likely scenario.

CNVs encompassing large genomic regions will potentially impair critical processes in spermatogenesis –mitosis required for the efficient proliferation and differentiation of spermatogonia, and the quality of meiosis resulting in haploid spermatids. There is an abundance of literature showing that large duplications affect meiotic chromosome pairing and lead to non-allelic homologous recombination events, generating genomic rearrangements^47,48^. Meiosis may be further impaired due to translocated duplications. Acrocentric segmental duplications promoting interchromosomal duplications between acrocentric and non-acrocentric chromosomes 1, 3, 4, 7, 9, 16, and 20 are particularly prone to double-strand breaks^49^. Large CNVs in pericentromeric regions identified on chromosomes 14, 15, 16, and 19 may modulate the stability of long stretches of constitutive heterochromatin (Table 3, Supplementary Table S2), potentially affecting meiotic chromosome pairing and assembly of the kinetochore complex for chromosomal segregation^50,51^. Other reports on SPGF cases have also described CNVs in pericentromeric regions on chromosomes 13–16^52^. In our study, extensive deletions at 16p11.2 were detected in 12 patients with SPGF but not in fertile men. Chromosome 16 is susceptible to heteromorphisms and includes a hotspot for inversions leading to subsequent deletions or duplications that may predispose to errors in chromosomal segregation^53^. Reduced dosage of six testis-enriched *TP53TG3* genes in the deleted region may also contribute to SPGF. The role of structural variation at 16p11.2 in SPGF must be further studied using targeted long-read sequencing.

It has been discussed that there is an abundance of genomic rearrangement hotspots that harbor genes important for spermatogenesis^54^. Also in this study, novel candidate genes for male infertility were identified that were disrupted by CNVs (Table 1, Fig. 2). As a secondary outcome, a recurrent deletion within the testis-specific *LRRC69* gene was identified as a novel candidate risk factor for male infertility. It was carried by seven cases with SPGF (and no NORM) and presented significantly higher prevalence among infertile men than in the general Estonian population (3.3% vs. 0.9%). A possible link to spermatogenic parameters might be through carnitine levels ^55^, which are lowered by *LRRC69* loss-of-function variants^56^. Further investigation of the functional link between *LRRC69* and spermatogenesis is warranted.

Several large microdeletions and microduplications identified in this study have been mainly or only described in pediatric patients presenting ID/DD (Table 3). Alternative scenarios need to be considered to understand the pleiotropic effect of large CNVs in causing developmental disorders and/or potentially leading to SPGF. Whereas microdeletion/microduplication syndromes are mainly considered to be caused by altered dosage of critical genes, most highlighted CNVs did not encompass any apparent candidate genes for SPGF. As discussed above, the presence of a large genomic rearrangement per se may affect the complex process of spermatogenesis. In other occasions, large CNVs may involve genes implicated in brain development and function, as well as those required for spermatogenesis. Altered dosage of those genes will have differential effect, depending on the tissue of action. Notably, according to the Human Protein Atlas, the human brain and testis share the highest number of group enriched genes, indicating potential shared pathways in spermatogenesis and brain development and function^41^. One example is *PTPN20* (10q11.22 microduplication) that has the highest expression in both, testicular and brain tissues. It has been proposed that the role of these two tissues in the speciation process could explain the high similarity of their proteomic profile^57^. However, at present the clinical relevance (shared cause for neurological disorders and impaired spermatogenesis) of this topic is underexplored.

As a practical implication, the study data provided supportive evidence that introducing chromosomal microarray analysis into routine andrological workup of certain subgroups of cases with SPGF will improve their molecular diagnostics and clinical management. This additional genomic analysis will be relevant for idiopathic NOA and SO patients after standard genetic evaluation. Infertile men with sperm concentration less than 5 × 10^6^/ml could be tested, concordant with current recommendations for the analysis of Y chromosome *AZF* microdeletions^58^. Before genetic testing, counselling by a clinical geneticist could be considered for extended patient phenotyping and compilation of family health history. This advanced genetic assessment will facilitate not only evidence-based counselling of the couple about their reproductive choices and predisposition to pregnancy failure^18,59^ but also identification of congenital risks for health conditions of the patient and future offspring.

### Limitations

The limitations of the study have to be acknowledged. Standard chromosome analysis was not performed in 48 (22.3%) patients with SPGF and all individuals in the NORM group. As microarray-based analysis does not enable to detect inversions, translocations, and complex genomic rearrangements., these variant types may have been missed. Additionally, the exact breakpoints of deletions and duplications cannot be precisely determined with the resolution of the SNP microarray dataset. Also, this study did not analyze CNVs on the Y chromosome due to high level of genomic complexity and low number of unique SNPs. Calling of Y-linked CNVs requires targeted genotyping and/or sequencing approaches^4^. Due to different approaches for CNV calling, only deletions/duplications of 10 kb or more were evaluated for the X chromosome and the load of CNVs on autosomes and the X chromosome could not be directly compared.

Recurrent disease-associated rare genetic variants identified in one population, such as the partial deletion of *LRRC69* in this study, may not be present in subjects with other ancestries. In the current retrospective study, health data and blood samples of family members had not been collected at patient recruitment. Therefore, the origin of the identified CNVs (de novo or inherited) could not be determined and the phenotypes of other possible CNV carriers in the family were unavailable. In perspective, collecting parental informed consent for their carrier testing and family history of health conditions should be recommended upon recruitment of patients with SPGF for genetic testing and research.

## Conclusions

The diagnostic pipeline of SPGF cases will benefit from chromosomal microarray analysis by identifying undiagnosed carriers of clinically relevant microdeletions and microduplications. This will add significant value in the routine management and counselling of infertile men for their general and reproductive health.

## Supplementary Information

Supplementary Information 1.

Supplementary Information 2.

## Data Availability

All data generated or analyzed during this study are included in this published article. Data of the identified CNVs have also been submitted to NCBI dbVar (https://www.ncbi.nlm.nih.gov/dbvar/) under the study accession number nstd227.
