# Supplementary material for: Microdeletions and microduplications linked to severe congenital disorders in infertile men
Source: Sci Rep. 2023 Jan 11;13:574. doi: 10.1038/s41598-023-27750-w (PMC9834233; doi:10.1038/s41598-023-27750-w)
Supplement: Supplementary file 1 — Supplementary Information 1. [file 41598_2023_27750_MOESM1_ESM.docx]

**Microdeletions and microduplications linked to severe congenital disorders in infertile men**

Triin Kikas^1^; Anna Maria Punab^1^; Laura Kasak^1^; Olev Poolamets^2^; Vladimir Vihljajev^2^; Kristjan Pomm^2^; Mario Reiman^1^; Stanislav Tjagur^2^; Paul Korrovits^2^; Margus Punab ^1,2,3^; Maris Laan^1^*.

^1^ Chair of Human Genetics, Institute of Biomedicine and Translational Medicine, University of Tartu, Tartu 50411, Estonia

^2^ Andrology Clinic, Tartu University Hospital, Tartu 50406, Estonia

^3^ Institute of Clinical Medicine, University of Tartu, Tartu 50406, Estonia

* Correspondence:

Maris Laan, PhD, Chair of Human Genetics, Institute of Biomedicine and Translational Medicine, University of Tartu, Ravila 19, Tartu 50411, Estonia. Phone: +372-7375008; E-mail: [maris.laan@ut.ee](mailto:maris.laan@ut.ee)

**Supplementary Methods**

**Recruitment and andrological phenotyping**: Study participants were recruited to the Estonian Andrology (ESTAND) cohort^[1]^ and the material was collected at the Andrology Clinic, Tartu University Hospital (AC-TUH), Estonia. AC-TUH represents the primary and referral center managing >90% of all male infertility cases in the country. All 277 study subjects had undergone routine andrological workup^[1]^ by andrology specialists using the established andrological pipeline and standard protocols at the Andrology Centre, Tartu University Hospital (AC-TUH). All andrologists had previously received training in standardized clinical assessment in European Andrology Academy accredited centers, including AC-TUH. Couple infertility was diagnosed as a failure to conceive a child for a period of ≥12 months.

Physical examination included the assessment of genital phenotype and testicular size with an orchidometer (birch wood, Pharmacia and Upjohn, Denmark) in a standing position. Details for the total testis volume (sum of left and right testicles), position of the testicles in scrotum, pathologies of the genital ducts, penis, urethra and presence and grade of varicocele were registered for each patient. Sperm analyses were conducted according to the World Health Organization (WHO)^[2]^ recommendations on samples acquired by patient masturbation. The semen samples were incubated at 37°C for 30-40 minutes for liquefaction. Then, the volume was estimated by weight, subtracting the collection tube weight and assuming 1 g = 1 ml. Spermatozoa concentration was estimated on diluted samples (with 0.6 mol/l NaHCO3%, 0.4% (v/v) formaldehyde in distilled water) using the improved Neubauer hemocytometers.

Spermatogenic failure (SPGF, total sperm count <39×10^6^ per ejaculate) was defined according to the World Health Organization guidelines^[2]^. Stratification to severe (SO, sperm count >0 and <10×10^6^) and moderate oligozoospermia (MO, 10-39×10^6^) was implemented as described^[1]^. Non-obstructive azoospermia (NOA) refers to a lack of sperm in the ejaculate due to primary SPGF.

**Study group formation:** Genome-wide CNV profile was generated and analyzed for 277 ESTAND participants (median 33, 18-58 years; **Table 1**). All participants were of white European ancestry and living in Estonia. Patients with SPGF (total, n=215) were selected from the dataset of a 9-year (2005-2013) prospective monocenter study of 1,737 men with reduced sperm count^[1]^. In this published study, the primary cause of infertility had been defined for 695, whereas 1,042 cases remained unexplained. As the current investigation assumed that CNVs may represent genetic factors causing or predisposing to SPGF, we focused on more extreme idiopathic cases. All 67 unexplained NOA cases from 2005-2013 plus six additional NOA cases from 2004 were included (total NOA subgroup, n=73). The subgroups of 86 SO and 58 MO cases were formed from idiopathic patients with the DNA available at the time of genotyping and attempting to match the general parameters of the NOA subgroup. In addition, 62 normozoospermic fertile men with all andrological parameters in the normal range (sperm count, testicular volume, reproductive hormones) were used as controls (NORM subgroup) for the current study. Originally, these men had been recruited to the study of fertile partners of pregnant women, passing identical andrological workup at AC-TUH as infertile men^[1],[3]^.

Cytogenetic karyotyping analysis was missing for 48 patients with SPGF (n=11 NOA, 14 SO, 23 MO) and all men in the NORM subgroup. Therefore, all samples in this study were evaluated for sex chromosomal abnormalities using the genome-wide SNP genotyping data and the established approach^[4]^.

Height and weight had not been measured for 22 study subjects, semen volume had not been documented in seven, and serum testosterone level for one man. As these parameters were not included in the study inclusion and subgroup formation criteria, the missing information did not affect the core outcomes and conclusions.

**Patient exclusion criteria:** Patients diagnosed with known genetic and non-genetic causal factors for male infertility^[1],[2]^, such as secondary hypogonadism, seminal tract obstruction, cytogenetic abnormalities, Y-chromosomal microdeletions, cryptorchidism, sexual dysfunction, androgen abuse, severe traumas and operations in genital area or chemo- or radiotherapy were excluded from this study.

**Genome-wide SNP genotyping**: Blood genomic DNA of all samples was genotyped using Illumina HumanOmniExpress-24-v1.0/v1.1 BeadChips at the institutional genotyping core facility (UT, Institute of Genomics, Core Facility of Genomics; https://genomics.ut.ee/en/genomics-core-facility). Samples were genotyped with an average overall call rate of 99.9% (median 99.9%). Quality control of SNP genotyping data followed published recommendations^[5]^. The study included only samples and SNPs with genotyping call rates >99% and >95%, respectively. In total 707,950 SNPs present at the same genomic location on both v1.0/v1.1 of HumanOmniExpress-24 genotyping arrays were used for CNV calling. Identity-by-descent analysis was implemented in PLINK ver 1.9^[6]^ to exclude any relatives among study cases.

**Detection of autosomal CNVs:** The pipeline applied for autosomal CNV calling based on Illumina HumanOmniExpress-24 SNP-array has been previously described^[7]^. For each sample, parallel analysis of CNVs with three algorithms was carried out. Normalized signal intensity data was obtained through Illumina GenomeStudio software v2.0.5. Information on Log2 R ratios, B allele frequencies, markers, and chromosomal coordinates from each sample were used for CNV identification with Hidden Markov Model-based algorithm QuantiSNP v2.3^[8]^ that has been optimized for the Illumina platform data, and a sparse Bayesian learning approach GADA (Genome Alteration Detection Analysis)^[9]^. For QuantiSNP and GADA, CNV calling was performed separately for each individual. QuantiSNP was run using the default calling parameters with the adjustment for ‘genomic waves’ in signal intensities using the ‘--doGCcorrect’ key. GADA was implemented using the options “aAlpha 0.2 -T 5 -M 3”. CNVs with QuantiSNP log Bayes Factor value < 10 or GADA segment mean amplitude -0.2 to 0.1 were excluded from the resulting lists of called CNVs entering the subsequent merging step. The CNstream^[10]^ algorithm that has been specifically designed for the Illumina SNP-arrays was run for all 277 samples simultaneously to estimate CNVs from X and Y channel intensities loaded from Illumina GenomeStudio (v2.0.5). CNstream was implemented with the following criteria: number of SNP probes defining a segment (=3); minimum number of scores in one segment exceeding the threshold for identifying an amplification/deletion (=3); CNV minimum frequency threshold (=0%), maximum segment length (=1000000 bp) and an optional normalization step to standardize the marker intensity distributions between experimental batches. Overlapping CNV segments called by CNstream for each individual were merged into one CNV.

The HD-CNV (Hotspot Detector for Copy Number Variants) algorithm^[11]^ was used to detect overlapping regions among calls by alternative computational algorithms^[7]^. A criterion of 40% reciprocal overlap (with a minimal length 100 bp) between parallel detection of the same type of CNV (deletion or duplication) was used to define two calls as identifying the same event. All CNVs called by at least two algorithms for the same individual in the same genomic loci were considered in the subsequent global analysis.

**Detection of X-linked CNVs:** The applied CNV calling tools developed for the detection of autosomal CNVs based on genome-wide SNP microarray data have been reported as unreliable in detecting true sex-chromosomal CNVs, especially for the heterogametic sex^[12],[13]^. It is noted in QuantiSNP program documentation that it is known to overestimate CNV calls on chromosome X on older versions of Illumina cluster files. High-resolution custom-design arrays are recommended as a preferred method to detect X-chromosomal CNVs linked to male infertility reliably^[14],[15]^.

In this study, X-chromosomal CNVs were called using the Illumina GenomeStudio 2.0.5 built-in CNV analysis tool (cnvPartition CNV Analysis Plugin v3.2.1) with default settings (confidence threshold =35, detect extended homozygosity =true, exclude intensity only =false, GC wave adjust =false, include sex chromosomes =true, minimum homozygous region size =1000000 bp, minimum probe count =3). To avoid spurious CNV predictions, only variants >10kb were considered for the subsequent analysis. The confidence of prediction for all included CNVs was confirmed by visual inspection of Log R ratio plots for the detected regions. We acknowledge that due to different CNV detection methods, the load of CNVs on the autosomes and the X-chromosome could not be directly compared. This study also did not analyze CNVs on the Y-chromosome due to high level of genomic complexity and low number of unique SNPs. Calling of Y-linked CNVs requires targeted genotyping and/or sequencing approaches^[16]^.

**Array-CGH:** Blind external validation of 7p22.3-p22.2 microduplication (Case SO1) was carried out by the commercial service provider Biomedicum Functional Genomics Unit (FuGU), Helsinki, Finland (https://www2.helsinki.fi/en/infrastructures/genome-analysis/infrastructures/biomedicum-functional-genomics-unit). CNV detection was performed using array comparative genomic hybridization (aCGH) implemented at the Agilent Human Genome CGH 2 x 400K SurePrint G3 Microarray platform (Agilent Technologies, Santa Clara, USA). The reference genomic DNA for aCGH represented an anonymous male DNA sample available by the service provider; array hybridizations and quality control followed the established pipeline. CNVs were called with the Agilent Genomic Workbench 7.0.4.0 software, setting the minimum absolute average Log Ratio to 0.25. The minimum number of probes called in the CNV region was 4.

**Taqman qPCR:** Recurrent deletion covering four exons of the *LRRC69* gene (overlapping region: chr8:92128840-92181214 (hg19)) was experimentally validated using TaqMan qPCR. A predesigned TaqMan copy number assay (Assay ID Hs03272098_cn) and protocols recommended by the manufacturer (Applied Biosystems, Foster City, CA, USA) were used. DNA copy number typing was performed using HOT FIREPol Probe qPCR Mix (Solis BioDyne OÜ, Tartu, Estonia) in duplex reactions including both, the tested and the reference locus (TaqMan® Copy Number Reference Assay, human, RNase P). Copy number was normalized to the reference of RNase P and to the custom population-specific pool of randomly selected 50 fertile control DNAs. All reactions were run in triplicate and detected on ABI Prism 7900HT Sequence Detection System (Applied Biosystems).

**The Estonian biobank (EstBB):** EstBB is a population-based biobank established in 2002^[17]^. All EstBB participants have been genotyped at the Core Genotyping Lab of the Institute of Genomics, University of Tartu, using Illumina Global Screening Array v1.0 and v2.0. A subset of the EstBB dataset has been utilized for genome-wide CNV detection^[18],[19]^. In this study, data from 45,390 EstBB individuals was available to estimate the prevalence of the *LRRC69* intragenic deletion in the Estonian population.


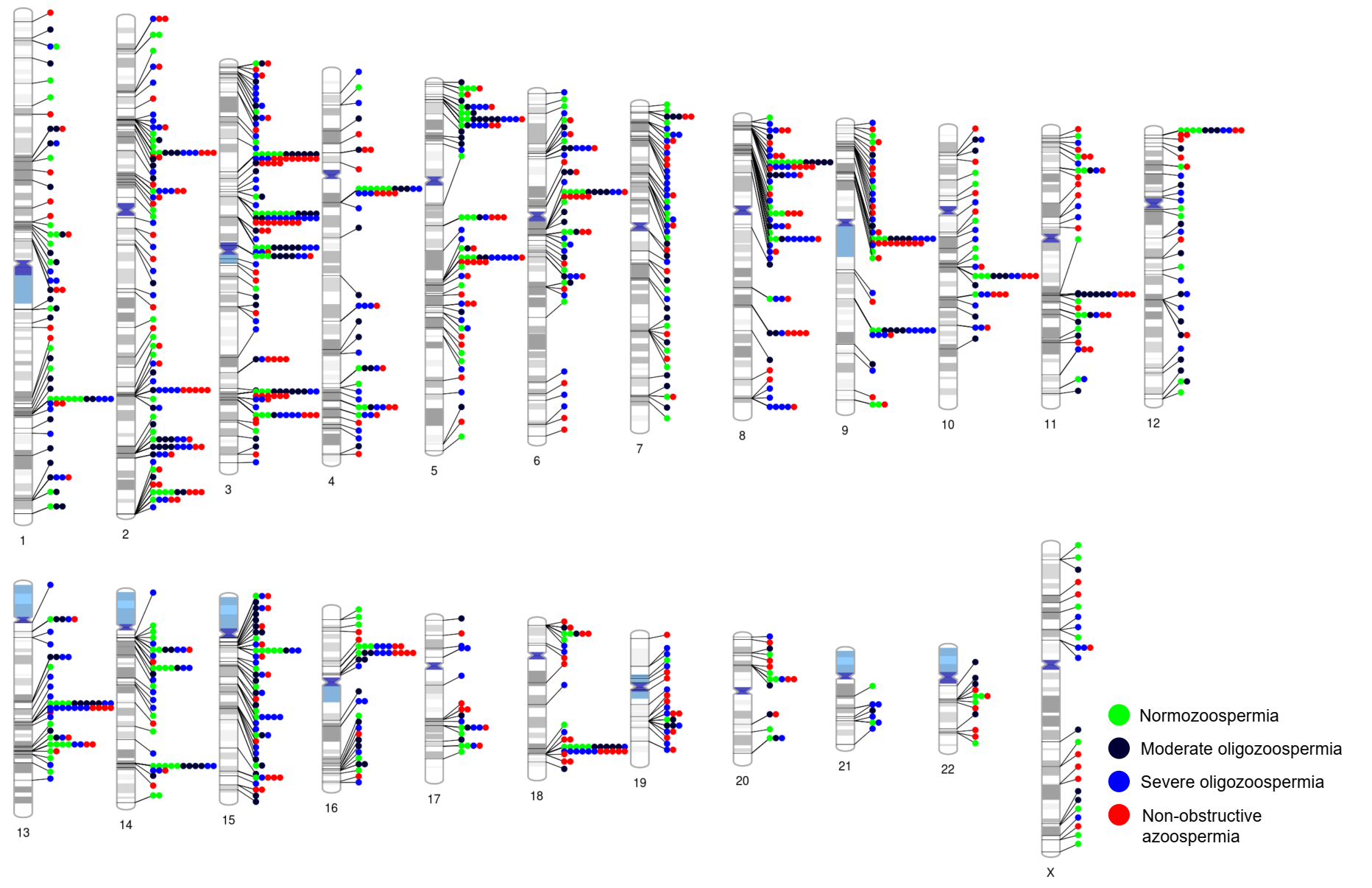


Supplementary Figure S1. Chromosomal distribution of the identified deletions in 277 study subjects.

Patients were stratified to groups based on their sperm count: non-obstructive azoospermia (no sperm in the ejaculate), severe (sperm count >0 and <10×10^6^) and moderate oligozoospermia (10-39×10^6^). Normozoospermia refers to total sperm count ≥39×10^6^/ejaculate.


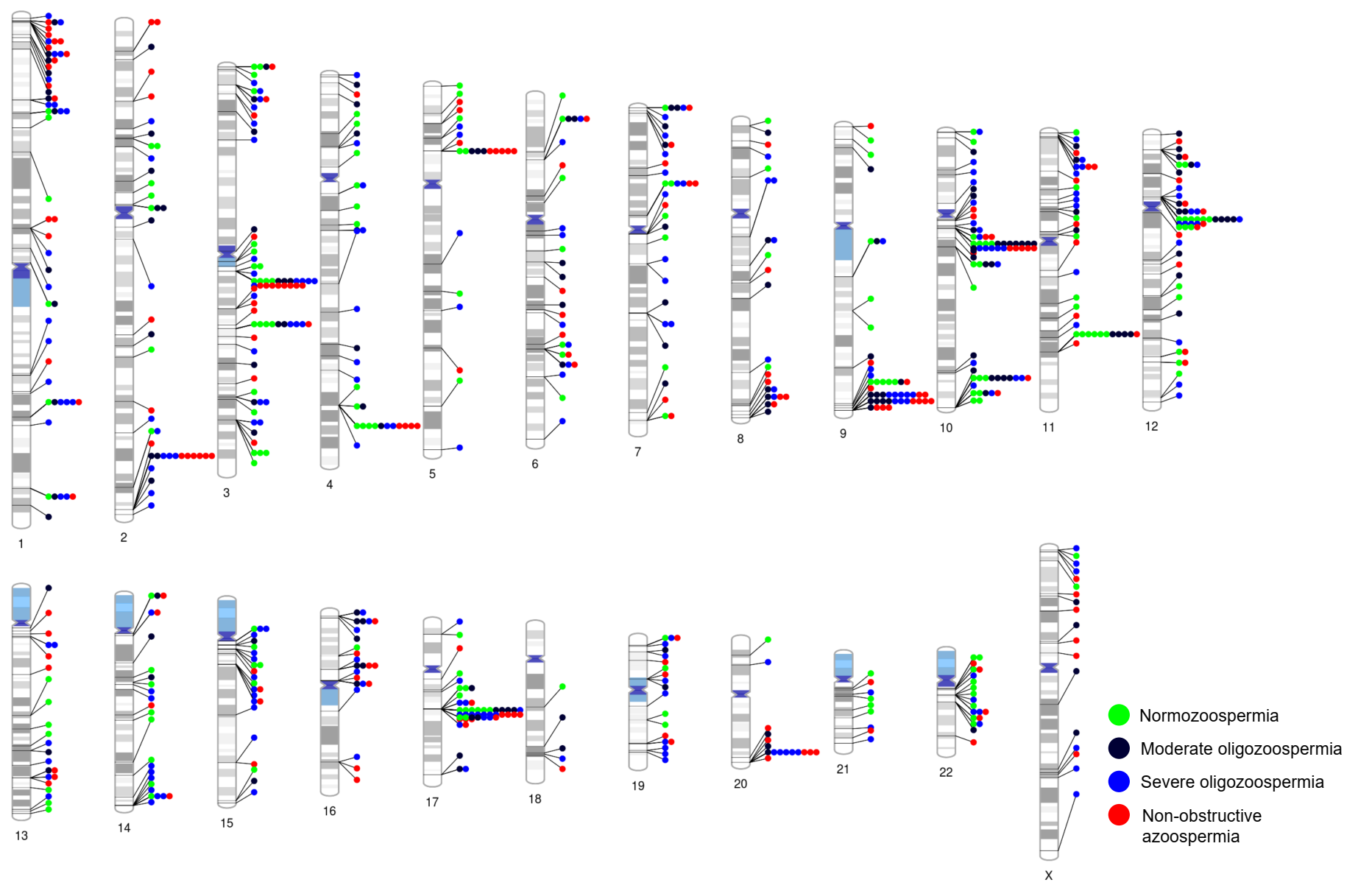


Supplementary Figure S2. Chromosomal distribution of the identified duplications in 277 study subjects.

Patients were stratified to groups based on their sperm count: non-obstructive azoospermia (no sperm in the ejaculate), severe (sperm count >0 and <10×10^6^) and moderate oligozoospermia (10-39×10^6^). Normozoospermia refers to total sperm count ≥39×10^6^/ejaculate.


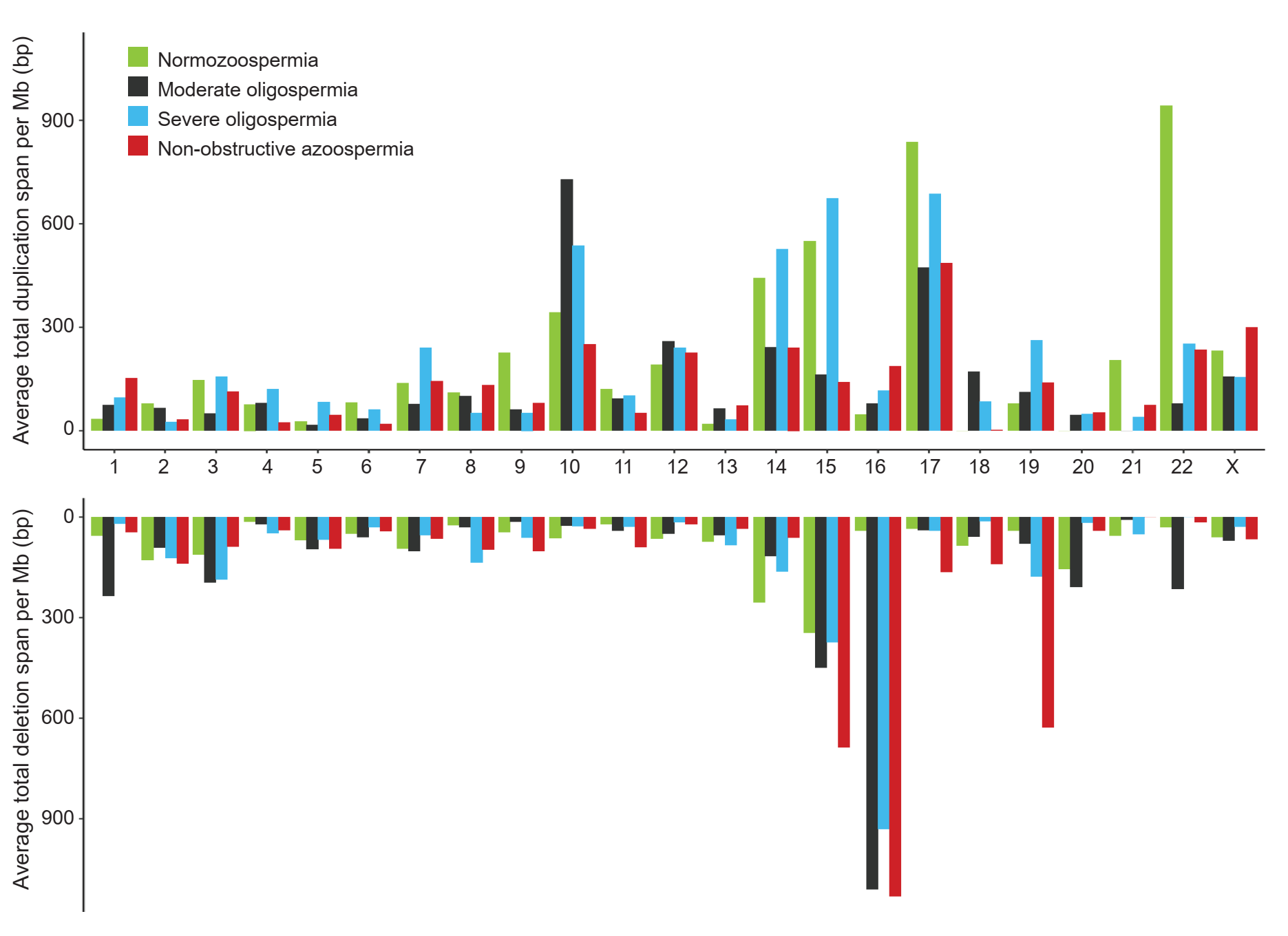


Supplementary Figure S3. Burden of deletions and duplications on each chromosome in the study subjects stratified for the spermatogenic output.

Patients were stratified to groups based on their sperm count: non-obstructive azoospermia (no sperm in the ejaculate), severe (sperm count >0 and <10×10^6^) and moderate oligozoospermia (10-39×10^6^). Normozoospermia refers to total sperm count ≥39×10^6^/ejaculate. Total burden of deletions and duplications on each chromosome was normalized per individual and per Mb.


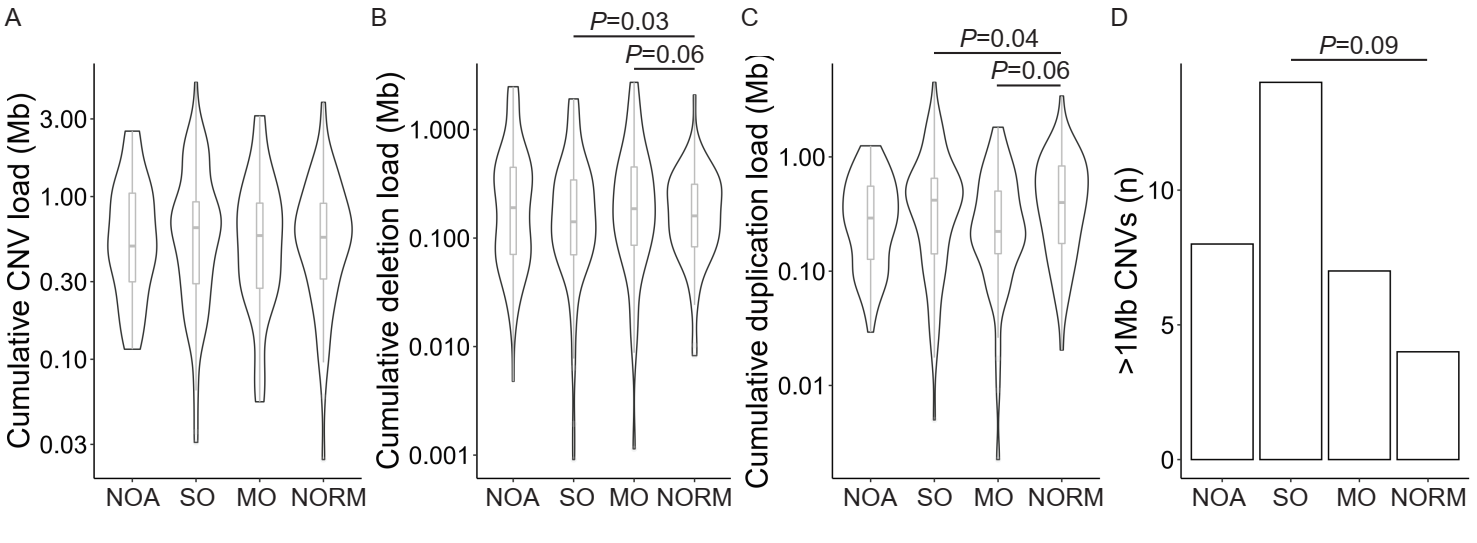


Supplementary Figure S4. Comparative summary statistics of the identified autosomal CNVs in the study subjects stratified for the spermatogenic output.

Panels A-C depict the distribution of cumulative CNV load with violin plots in black and boxplots in gray lines. Middle line of each corresponds to the median. Upper and lower sides of the box extend from the 25^th^ to 75^th^ percentiles, respectively (inter-quartile range, IQR). Whiskers are plotted according to the Tukey method, extending to the largest value no further than 1.5 times the IQR. Panel D shows the count of large CNVs detected in study subjects. For detailed data, please see **Table 2**.

NORM, normozoospermic controls (total sperm count ≥39×10^6^); MO, moderate oligozoospermia (10-39×10^6^); NOA, non-obstructive azoospermia (no sperm in the ejaculate); SO, severe oligospermia (>0 and <10×10^6^).


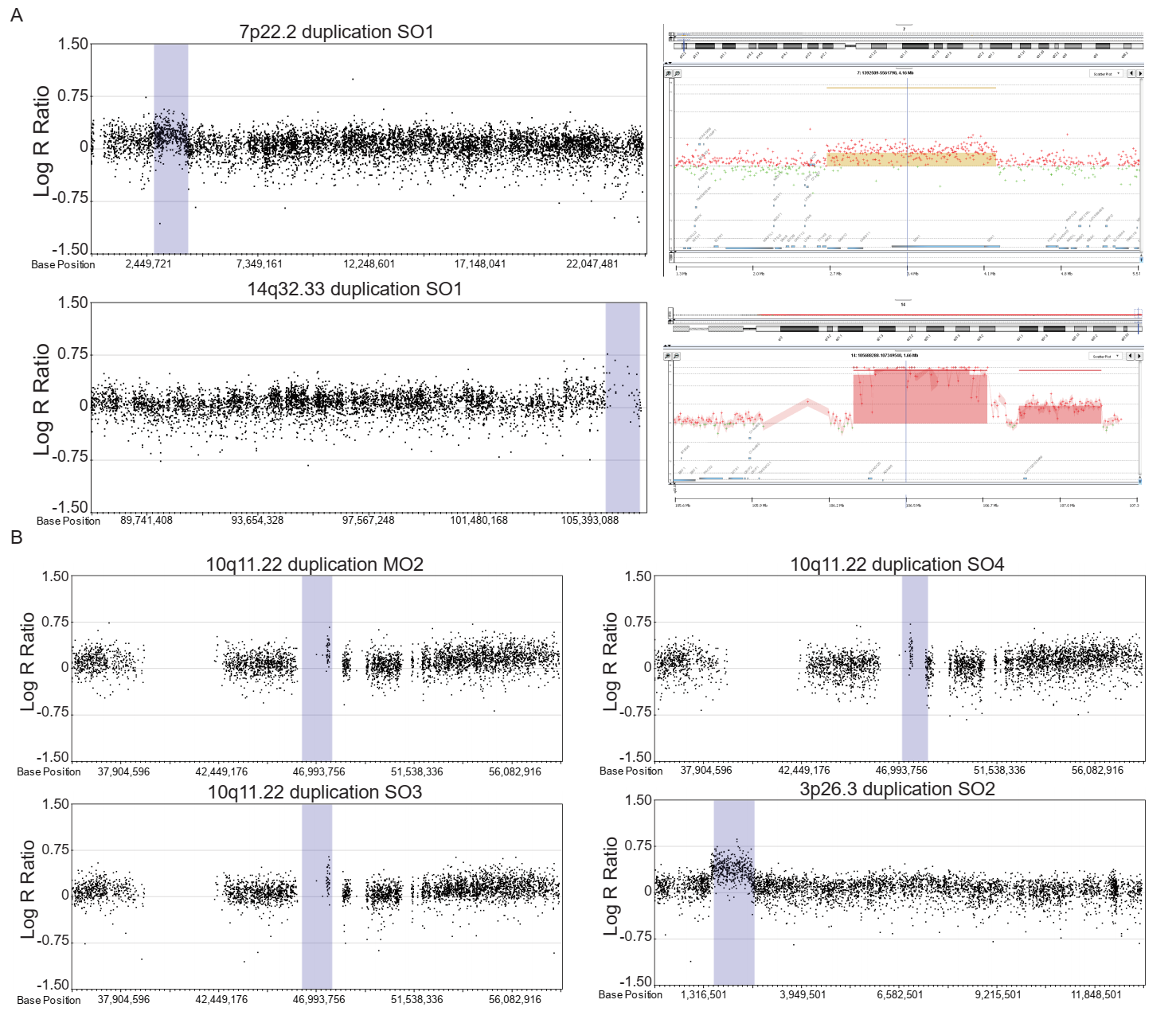


Supplementary Figure S5. Large duplications identified in male infertility patients SO1-SO4 and MO2.

**Panel A.** Case SO1 carried large duplications at 7p22.3-p22.2 (1.56 Mb) and 14q32.33 (1.22 Mb). These duplications are visualized using the output from GenomeStudio 2.0.5 CNV calling pipeline based on Illumina HumanOmniExpress-24v1.0a data (left) and Agilent Workbench CNV calling platform based on the Agilent Human Genome CGH 2 x 400K SurePrint G3 Microarray data (right). Genome-wide genotyping was carried out in institutional genotyping core facility, Core Facility of Genomics, Tartu, Estonia. For experimental validation purpose, array-CGH was outsourced to commercial service provider Biomedicum Functional Genomics Unit (FuGU), Helsinki, Finland.

**Panel B.** Microduplication at 10q11.22 was detected in patients MO2 and SO3 with SPGF (both 1.42 Mb) and SO4 (1.22 Mb), and microduplication at 3p26.3 (1.1 Mb) in case SO2. The duplications were visualized with GenomeStudio 2.0.5 implemented for Illumina HumanOmniExpress-24v1.0a (MO2, SO3, SO2) and Illumina HumanOmniExpress-24v1.1a (SO4) datasets.

Total sperm count per ejaculate of these cases were 0.2×10^6^ (SO1), 2.6×10^6^ (SO2), 2.4×10^6^ (SO3), 7.4 ×10^6^ (SO4) and 21.3 ×10^6^ (MO2). Detailed clinical characteristics are provided in **Table S1**. Moderate (MO) and severe (SO) oligozoospermia were defined based on total sperm count per ejaculate 10-39×10^6^ or >0 and <10×10^6^, respectively. On all images, the x-axis depicts the chromosomal coordinates based on hg37, and the y-axis indicates Log R ratio.

## References

1. Punab, M. *et al.* Causes of male infertility: a 9-year prospective monocentre study on 1737 patients with reduced total sperm counts. *Hum. Reprod.* **32**, 18–31 (2017).

2. WHO. World Health Organization. WHO laboratory manual for the examination and processing of human semen. 6th ed. World Health Organization, Department of Reproductive Health and Research. Geneva, Switzerland. *WHO Press* (2021).

3. Ehala-Aleksejev, K. & Punab, M. The effect of metabolic syndrome on male reproductive health: A cross-sectional study in a group of fertile men and male partners of infertile couples. *PLoS One* **13**, (2018).

4. Sanders, S. J. *et al.* Insights into Autism Spectrum Disorder Genomic Architecture and Biology from 71 Risk Loci. *Neuron* **87**, 1215 (2015).

5. Cooper, N. J. *et al.* Detection and correction of artefacts in estimation of rare copy number variants and analysis of rare deletions in type 1 diabetes. *Hum. Mol. Genet.* **24**, 1774 (2015).

6. Chang, C. C. *et al.* Second-generation PLINK: rising to the challenge of larger and richer datasets. *Gigascience* **4**, 7 (2015).

7. Kasak, L., Rull, K., Vaas, P., Teesalu, P. & Laan, M. Extensive load of somatic CNVs in the human placenta. *Sci. Rep.* **5**, 8342 (2015).

8. Colella, S. *et al.* QuantiSNP: an Objective Bayes Hidden-Markov Model to detect and accurately map copy number variation using SNP genotyping data. *Nucleic Acids Res.* **35**, 2013–2025 (2007).

9. Pique-Regi, R., Cáceres, A. & González, J. R. R-Gada: A fast and flexible pipeline for copy number analysis in association studies. *BMC Bioinformatics* **11**, 1–12 (2010).

10. Alonso, A. *et al.* CNstream: A method for the identification and genotyping of copy number polymorphisms using Illumina microarrays. *BMC Bioinformatics* **11**, 1–18 (2010).

11. Butler, J. L. *et al.* HD-CNV: hotspot detector for copy number variants. *Bioinforma. Appl.* **29**, 262–263 (2013).

12. Lopes, A. M. *et al.* Human Spermatogenic Failure Purges Deleterious Mutation Load from the Autosomes and Both Sex Chromosomes, including the Gene DMRT1. *PLoS Genet.* **9**, 1003349 (2013).

13. Teshiba, R. *et al.* Identification of TCTE3 as a gene responsible for congenital diaphragmatic hernia using a high-resolution single-nucleotide polymorphism array. *Pediatr. Surg. Int.* **27**, 193–198 (2011).

14. Vockel, M., Riera-Escamilla, A., Tüttelmann, F. & Krausz, C. The X chromosome and male infertility. *Hum. Genet.* **140**, 203–215 (2021).

15. Yatsenko, S. A., Wood-Trageser, M., Chu, T., Jiang, H. & Rajkovic, A. A high-resolution X chromosome copy-number variation map in fertile females and women with primary ovarian insufficiency. *Genet. Med.* **21**, 2275–2284 (2019).

16. Hallast, P. *et al.* A common 1.6 mb Y-chromosomal inversion predisposes to subsequent deletions and severe spermatogenic failure in humans. *Elife* **10**, (2021).

17. Leitsalu, L. *et al.* Cohort Profile: Estonian Biobank of the Estonian Genome Center, University of Tartu. *Int. J. Epidemiol.* **44**, 1137–1147 (2015).

18. Männik, K. *et al.* Copy Number Variations and Cognitive Phenotypes in Unselected Populations. *JAMA* **313**, 2044–2054 (2015).

19. Auwerx, C. *et al.* The individual and global impact of copy-number variants on complex human traits. *Am. J. Hum. Genet.* **109**, 647 (2022).
